# Integrated MicroRNA–mRNA Analysis Reveals miR-204 Inhibits Cell Proliferation in Gastric Cancer by Targeting *CKS1B*, *CXCL1* and *GPRC5A*

**DOI:** 10.3390/ijms19010087

**Published:** 2017-12-28

**Authors:** Sirjana Shrestha, Chi-Dung Yang, Hsiao-Chin Hong, Chih-Hung Chou, Chun-San Tai, Men-Yee Chiew, Wen-Liang Chen, Shun-Long Weng, Chung-Chu Chen, Yi-An Chang, Meng-Lin Lee, Wei-Yun Huang, Sheng-Da Hsu, Yi-Chang Chen, Hsien-Da Huang

**Affiliations:** 1 Institute of Bioinformatics and Systems Biology, National Chiao Tung University, Hsinchu 300, Taiwan; sirju10@yahoo.co.in (S.S.); chidung1212@gmail.com (C.-D.Y.); ezrachelkimo@gmail.com (H.-C.H.); chchou23@gmail.com (C.-H.C.); richard0609@gmail.com (W.-Y.H.); ken.bi95g@nctu.edu.tw (S.-D.H.); 2 Department of Biological Science and Technology, National Chiao Tung University, Hsinchu 300, Taiwan; chungsan0017019@gmail.com (C.-S.T.); chiewmene@gmail.com (M.-Y.C.); wenurea@yahoo.com.tw (W.-L.C.); b8703126@gmail.com (Y.-A.C.); martin25_lee@hotmail.com (M.-L.L.); 3 Institute of Population Health Sciences, National Health Research Institutes, Miaoli 350, Taiwan; 4 Institute of Molecular Medicine and Bioengineering, National Chiao Tung University, Hsinchu 300, Taiwan; cellbox@gmail.com; 5 Department of Medicine, Mackay Medical College, New Taipei City 252, Taiwan; a4467@ms7.mmh.org.tw; 6 Department of Obstetrics and Gynecology, Hsinchu Mackay Memorial Hospital, Hsinchu 300, Taiwan; 7 Mackay Junior College of Medicine, Nursing and Management College, Taipei 112, Taiwan; 8 Department of Medical Research, Hsinchu Mackay Memorial Hospital, Hsinchu 300, Taiwan; 9 Division of Hepatology and Gastroenterology, Department of Internal Medicine, Hsinchu Mackay Memorial Hospital, Hsinchu 300, Taiwan; a4059@icloud.com

**Keywords:** microRNA (miRNA), integrated analysis, gastric cancer, expression profiling

## Abstract

Gastric cancer (GC) is the second most frequent cause of cancer-related deaths worldwide. MicroRNAs are single-stranded RNA molecules of 21–23 nucleotides that regulate target gene expression through specific base-pairing interactions between miRNA and untranslated regions of targeted mRNAs. In this study, we generated a multistep approach for the integrated analysis of miRNA and mRNA expression. First, both miRNA and mRNA expression profiling datasets in gastric cancer from the Gene Expression Omnibus (GEO) and The Cancer Genome Atlas (TCGA) identified 79 and 1042 differentially expressed miRNAs and mRNAs, respectively, in gastric cancer. Second, inverse correlations between miRNA and mRNA expression levels identified 3206 miRNA–mRNA pairs combined with 79 dysregulated miRNAs and their 774 target mRNAs predicted by three prediction tools, miRanda, PITA, and RNAhybrid. Additionally, miR-204, which was found to be down-regulated in gastric cancer, was ectopically over-expressed in the AGS gastric cancer cell line and all down-regulated targets were identified by RNA sequencing (RNA-seq) analysis. Over-expression of miR-204 reduced the gastric cancer cell proliferation and suppressed the expression of three targets which were validated by qRT-PCR and luciferase assays. For the first time, we identified that *CKS1B*, *CXCL1*, and *GPRC5A* are putative targets of miR-204 and elucidated that miR-204 acted as potential tumor suppressor and, therefore, are useful as a promising therapeutic target for gastric cancer.

## 1. Introduction

Gastric cancer (GC) is the second leading cause of cancer-related deaths worldwide [1]. The major risk factors for the disease are dietary factors like consuming foods containing high salt, tobacco smoking, and *Helicobacter pylori* infection [2]. Activation of proto-oncogenes and inactivation of some tumor suppressor genes due to mutations also lead to gastric cancer [3]. Despite advances in detection and treatment approaches like surgery being effective in recent years, the five-year survival rate is less than 10% due to metastasis at the time of diagnosis [4].

MicroRNAs (miRNAs) are small non-coding RNA oligonucleotides approximately of 21–23 nucleotides that regulate genes at the post-transcriptional level. The regulation involves two different mechanisms either by suppression of mRNA translation or by induction of target mRNA cleavage [5]. miRNAs control more than half of the mammalian protein-coding genes [6]. MiRNAs are involved in different cellular processes like metabolism, differentiation, development and apoptosis and can furthermore regulate both oncogenes and tumor suppressor genes [7]. Aberrant miRNAs and mRNAs profiles have been noted in many cancers including gastric cancer [8,9,10,11,12]. As such, miRNA and mRNA expression should be subjected to integrated analysis to enhance our understanding of miRNAs and mRNAs in the process of tumorigenesis. Recently, many databases and target prediction tools have identified the potential targets of miRNAs. However, there is a significant challenge to identify the true targets of miRNAs. The effective targets of miRNAs that can act as biomarkers in gastric cancer should also be determined to develop therapeutic approaches with an enhanced efficiency. Although many studies have shown the dysregulation of miRNAs in gastric cancer, such a multistep approach for an integrated analysis has yet to be carried out to identify important miRNA and their targets as biomarkers in gastric cancer.

In this study, a multistep and systematic approach was generated for integrated analysis of miRNA and mRNA expression profiling. First, miRNA and mRNA expression profiling datasets in gastric cancer were collected from Gene Expression Omnibus (GEO) [13] and The Cancer Genome Atlas (TCGA) [14,15] and analyzed. Bioinformatics analysis identified 79 miRNAs and 1042 mRNAs that were differentially expressed in gastric cancer. Second, inverse correlations between miRNA expression and mRNA expression were applied. Third, we retained only those miRNAs and mRNAs target relationships which were predicted by three prediction tools, miRanda [16], PITA [17], and RNAhybrid [18]. A total of 3206 miRNA–mRNA pairs combined with 79 dysregulated miRNAs and their 774 target mRNAs were identified. Among the down-regulated miRNAs, miR-204 was selected for ectopic over-expression in the AGS gastric cancer cell line. MiR-204 was found to be deregulated in various cancers such as endometrial [19], ovarian, breast and renal cancers [20] and acts as tumor suppressor. Moreover, miR-204 was ectopically over -expressed in AGS cells which were then analyzed by subsequent RNA sequencing (RNA-seq) and all the down-regulated targets of miR-204 were identified. Both the prediction and RNA-seq analysis identified five candidate targets of miR-204, *CD55*, *CKS1B*, *CXCL1*, *GPRC5A* and *TNS4*, which were validated using qRT-PCR analysis. Furthermore, the luciferase reporter assay also identified *CKS1B*, *CXCL1*, and *GPRC5A* as putative targets of miR-204. Also, cell proliferation assays showed that miR-204 could suppress the growth of gastric cancer cell proliferation. Finally, gene ontology analysis revealed that miRNA-regulated genes have a potential role in biological processes such as regulation of cell cycle, regulation of apoptosis, programmed cell death, acute inflammatory response, regulation of cell proliferation and signal transduction.

## 2. Results

### 2.1. Differentially Expressed mRNA and miRNA in Gastric Cancer Versus Normal Gastric Tissues

**Figure 1:**
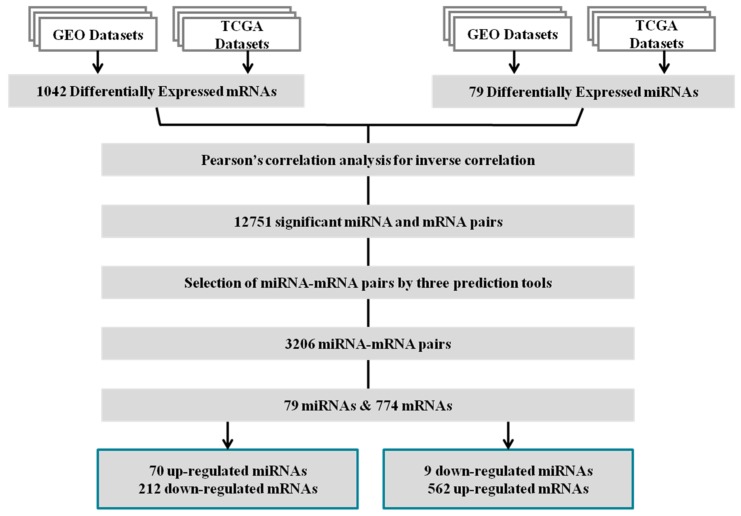
Flow chart for identifying differentially expressed miRNAs and mRNAs.

A systematic multistep approach combining miRNA and mRNA expression profiles and bioinformatics analysis was adopted to identify the GC-specific miRNA and mRNA interactions (Figure 1). To analyze the differentially expressed genes in GC, we collected four data sets (GSE13911, GSE19826, GSE17187 and GSE22377) deposited in GEO under platform GPL570 and TCGA Stomach Adenocarcinoma (STAD) datasets. The gene expression in normal and cancer tissues was statistically analyzed using Student’s *t*-test. A total of 2575 with 1830 up-regulated (Additional file 1: Table S1) and 745 down-regulated (Additional file 1: Table S2) genes were differentially expressed in GEO and 5967 with 4750 up-regulated (Additional file 1: Table S3) and 1217 down-regulated (Additional file 1: Table S4) genes were differentially expressed in TCGA STAD datasets.

**Figure 2:**
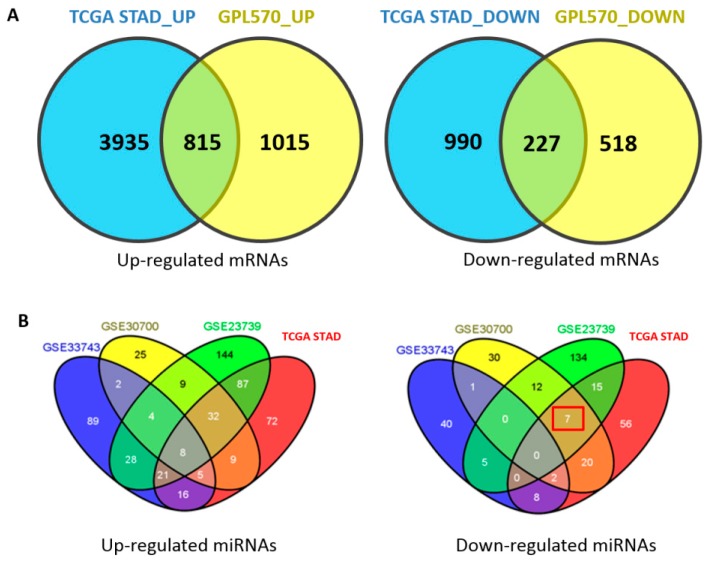
Differentially expressed genes and miRNAs in gastric cancer. (**A**) Up-regulated and down-regulated mRNAs. (**B**) Up-regulated and down-regulated miRNAs. A large set (shown in red box) which included GSE30700/GSE23739/TCGA STAD has 7 miRNAs.

The intersection of the GPL570 and TCGA STAD datasets yielded 1042 differentially expressed genes of which 815 were up-regulated and 227 were down-regulated (Figure 2A & Additional file 1: Table S5).

Three public miRNA datasets deposited in GEO under accession numbers GSE33743, GSE30070, and GSE23739 and high-throughput sequencing datasets of TCGA STAD were collected. The list of differentially expressed miRNAs from GEO and TCGA STAD datasets is shown in Table 1 and Figure 2B. The detailed information of the differentially expressed miRNAs is provided in Additional file 2: Tables S1–S8. The intersection of these GEO datasets and TCGA STAD datasets including at least three datasets yielded 79 differentially expressed miRNAs. Of these miRNAs, 70 were up-regulated and nine were down-regulated (Figure 2B & Additional file 2: Table S9).

### 2.2. Integrated Analysis of miRNA and mRNA through Pearson’s Correlation Analysis and Target Prediction

Pearson’s correlation analysis was performed to examine the 79 differentially expressed miRNAs and 1042 differentially expressed mRNAs. Since miRNAs act as negative regulators, up-regulated miRNAs resulted in down-regulated target mRNAs, and vice versa. Four target prediction tools; miRanda, PITA, RNAhybrid and TargetScan were used to find the potential miRNAs and mRNA interactions. A total of 12,751 miRNA–mRNA pairs were obtained by the combination of correlation analysis and target predictions (Additional file 3: Table S1). However, for further analysis, only those interactions which were predicted by three prediction tools miRanda, PITA and RNAhybrid were retained. Thus, 3206 miRNA–mRNA pairs generated by 79 dysregulated miRNAs and their 774 target mRNAs were obtained. These 3206-predicted miRNA–mRNA pairs contained 70 up-regulated miRNAs targeting 212 down-regulated mRNAs and nine down-regulated miRNAs targeting 562 up-regulated mRNAs (Figure 1). All these 3206 interactions between down-regulated miRNAs and up-regulated mRNAs and vice versa are shown in Additional file 3: Table S2.

### 2.3. Functional Enrichment Analysis of the Predicted Target Genes

The miRNA target genes predicted by three prediction tools, miRanda, PITA and RNAhybrid, were analyzed using the functional annotation tool, Database for Annotation, Visualization and Integrated Discovery (DAVID). The results revealed that a significant number of target genes were found in categories such as cell cycle, cell proliferation, programmed cell death, regulation of cell cycle, regulation of cell proliferation, cell adhesion and immune response (Additional file 4: Table S1).

### 2.4. qRT-PCR Analysis of Down-Regulated miRNAs in Gastric Cancer and Candidate miRNA Selection for Experimental Validation

**Figure 3:**
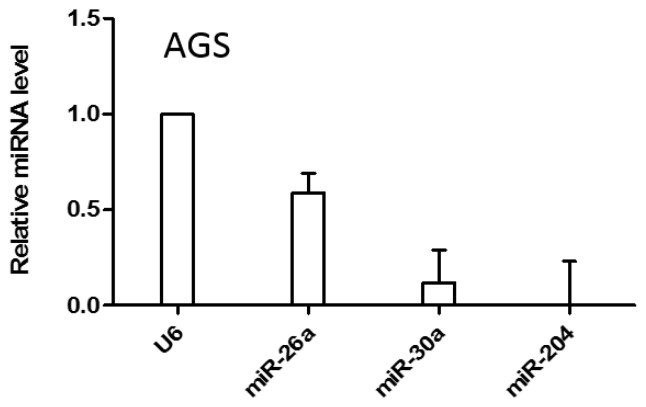
Relative miRNA expression in the AGS cell line. miRNA expression level was normalized to U6 miRNA and calculated from the triplicates data.

Of the 79 differentially expressed miRNAs, 70 were up-regulated and nine were down-regulated at least from three datasets. We focused on the down-regulated miRNAs because they can act as tumor suppressors in cancer and participate as therapeutic agents. For the preliminary screening of candidate miRNAs, three miRNAs (namely miR-204, miR-26a and miR-30a) were selected from the nine down-regulated miRNAs and subjected to experimental validation through an extensive literature search. The expression profile of these miRNAs in the AGS cell line was quantified using qRT-PCR. The miRNA expression level was normalized to U6 miRNA and miR-204 expression was found to be most down-regulated among the miRNAs in the AGS cell line (Figure 3). Furthermore, miR-204 was found to be most significantly down-regulated miRNA in two data sets among the large set, which included GSE30700/GSE23739/TCGA STAD with 7 miRNAs and small set, which comprised GSE33743/GSE30700/TCGA STAD with two miRNAs (Table 2). Thus, miR-204 was selected for further experimental validation. To further confirm whether miR-204 was inhibited in other gastric-cancer-associated cell lines, we measured the expression level of miR-204 in KATO III and SNU1 cell lines, and our results were consistent with those observed in the AGS cell line (Figure S1).

### 2.5. miR-204 Inhibits the Proliferation of Gastric Cancer Cells In Vitro

**Figure 4:**
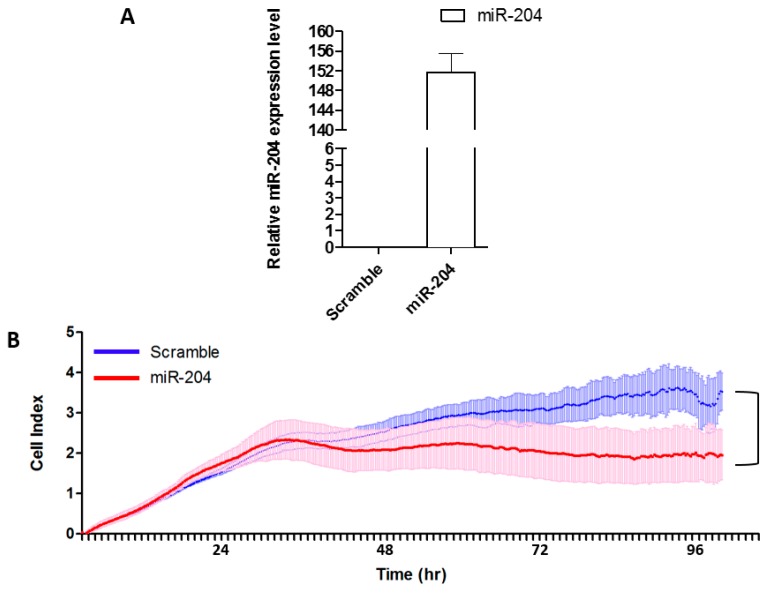
MiR-204 inhibits the proliferation of gastric cancer cells. (**A**) Over-expression of miR-204 was determined by real time qRT-PCR in AGS cells, (**B**) Cell proliferation was determined in the AGS gastric cancer cell line transfected with miR-204 mimic or scrambled control.

To determine the functional significance of miR-204 over-expression in gastric cancer cell proliferation, we transiently transfected the AGS gastric cancer cells with miR-204 mimic or scrambled control. The results showed that miR-204 mimic could drastically increase the level of miR-204 in AGS cells as shown in Figure 4A. For cell proliferation analysis the cell growth of AGS cells was monitored by using RTCA DP instrument. As shown in Figure 4B, transfection with miR-204 mimic suppressed the cell proliferation of gastric cancer cells 24-h post transfection compared with the transfection with scrambled control. This result suggested that miR-204 inhibited the proliferation of gastric cancer cells in vitro.

### 2.6. RNA-seq Analysis and Functional Enrichment of Down-Regulated Targets of miR-204

To profile the down-regulated target genes when miR-204 was over-expressed in the AGS gastric cancer cell line, we conducted RNA-seq analysis and adjusted the filter criteria with log2 fold change ≥0.5849 or ≤0.5849. RNA-seq analysis revealed 3233 differentially expressed target genes of which 1699 were up-regulated (Additional file 5: Table S1) and 1534 were down-regulated (Additional file 5: Table S2).

All of the down-regulated targets from RNA-seq analysis were applied to DAVID after miR-204 was over-expressed. The important gene ontology terms derived from the analysis in DAVID were cell cycle, cell proliferation and apoptosis (Table 3).

### 2.7. Candidate Target Genes of miR-204 for Experimental Validation

For the selection of candidate target genes of miR-204 for experimental validation out of all of the miR-204 target genes predicted by miRanda, PITA and RNAhybrid, and all of the down-regulated target genes from RNA-seq analysis after miR-204 over-expression in gastric cancer cells were intersected. The intersection resulted in 29 common target genes (Figure S2). However, all these miR-204 target genes remain unannotated in miRTarBase [21] (Additional file 6: Table S1), so these target genes were potential candidates for experimental validation. Five target genes, namely *CD55*, *CKS1B*, *CXCL1*, *GPRC5A* and *TNS4*, with significant gene ontology terms such as regulation of cell cycle, cell proliferation, cell death, cell cycle, programmed cell death, regulation of apoptosis, anti-apoptosis, signal transduction and immune response were selected for further experimental validation. The gene ontology of the selected target genes is as shown in Table 4.

### 2.8. miR-204 Suppresses the Expression of Multiple Target Genes in Gastric Cancer

**Figure 5:**
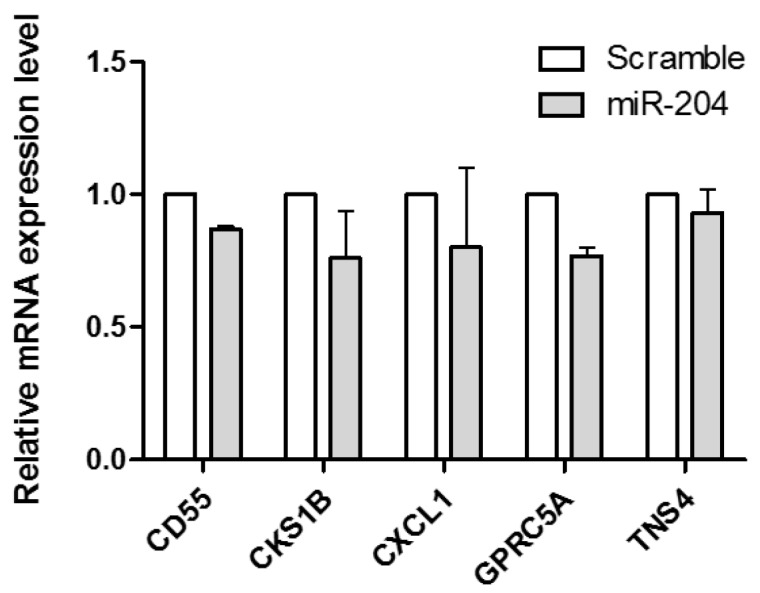
QRT-PCR validation of five target genes *CD55*, *CKS1B*, *CXCL1*, *GPRC5A* and *TNS4* of miR-204 common in RNA-seq analysis and prediction.

To investigate the regulation of target genes by miR-204, we over-expressed miR-204 and measured the expression levels of *CD55*, *CKS1B*, *CXCL1*, *GPRC5A* and *TNS4* by qRT-PCR. Before qRT-PCR, the AGS cells were transfected with miR-204 mimic and scrambled control. After 48 h of transfection, total RNA was isolated from AGS cells and the mRNA expression levels of five target genes were measured. Our qRT-PCR analysis confirmed that mRNA expression of these five target genes were down-regulated by miR-204 mimic in comparison to scrambled control as shown in Figure 5.

### 2.9. miR-204 Targets CKS1B, CXCL1, and GPRC5A in Gastric Cancer Cells

**Figure 6:**
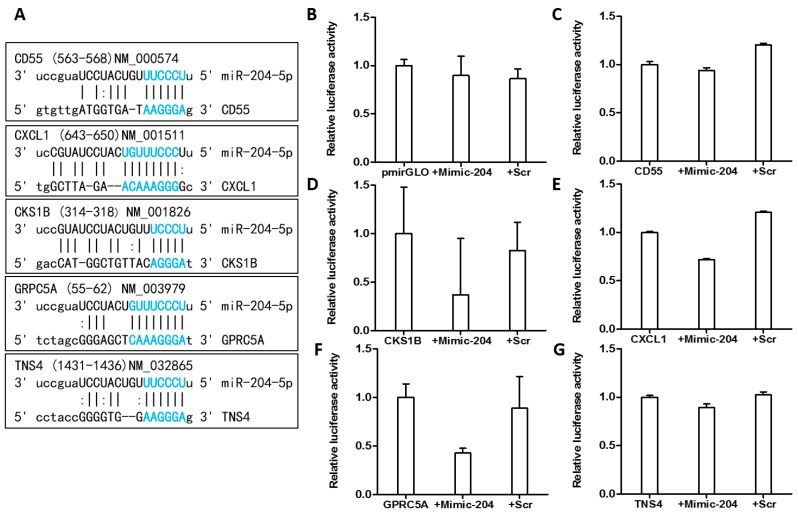
MiR-204 targets *CKS1B*, *CXCL1*, and *GPRC5A* in gastric cancer cells. (**A**) Predicted target sites (shown in blue) of miR-204 target genes. All the target regions were predicted by miRanda, PITA and RNAhybrid. Luciferase activity in AGS cells co-transfected with either (**B**) empty luciferase vector pmiRGLO as positive control or the construct containing 3′UTR of five target genes (**C**) *CD55*, (**D**) *CKS1B*, (**E**) *CXCL1*, (**F**) *GPRC5A* and (**G**) *TNS4* and miR-204 mimic (mimic) or scrambled control (scr).

MiRanda, PITA and RNAhybrid were used to identify the potential target sites of miR-204 and to understand its molecular mechanism as a tumor suppressor in GC. The potential target sites of miR-204 in *CD55*, *CKS1B*, *CXCL1*, *GPRC5A* and *TNS4* are as shown in Figure 6A.

To determine whether miR-204 can repress *CD55*, *CKS1B*, *CXCL1*, *GPRC5A* and *TNS4* by targeting its binding site at 3′UTR, we inserted the PCR product containing the full length of 3′UTR of these target genes into the pmiRGLO luciferase reporter vector. Considering that the translations of target genes are affected by miRNA, we performed dual luciferase reporter assays with AGS cells co-transfected with either empty luciferase vector or the construct containing 3′UTR of five target genes individually and with miR-204 mimic or scrambled control. The luciferase assay data indicated that the luciferase reporter activities of *CKS1B* (60%, Figure 6D) *CXCL1* (30%, Figure 6E), and *GPRC5A* (60%, Figure 6F) were markedly repressed by miR-204 mimic in comparison to scrambled control. The repression using the luciferase reporter assay might be due to binding of miR-204 to the 3′UTR of these target genes. However, such reduction was not observed in pmiRGLO no insert control (Figure 6B), *CD55* (Figure 6C), and *TNS4* (Figure 6G). These results suggest that miR-204 might target *CKS1B*, *CXCL1*, and *GPRC5A* via their 3′UTR.

## 3. Discussion

The potential use of miRNAs as a useful biomarker in diagnosis, prognosis and therapeutics has been increased tremendously in a short period; miRNAs act as negative regulators of gene expression by regulating different biological processes via inhibiting the expression of their target genes. Recently, many studies showed that miRNAs play critical roles in causing tumorigenesis of gastric cancer and have clinical significance in prognosis, diagnosis and treatment [22,23,24].

Here, a multistep approach was applied to reveal some miRNAs that are aberrantly expressed in gastric cancer and some of the miRNAs, and their target genes identified through integrated analysis were selected for experimental validation to show that miRNAs regulate their expression. This study focuses on the integrative analysis of gene and miRNA expression in gastric cancer. We used the TCGA STAD datasets and integrated the different sources of a cohort study from the public GEO database. We also performed fold change and statistical t-test to select the differentially expressed genes across the different datasets. However, extensive genetic and phenotypic variations are well known to exist between tumors [25], and the differences between genomes of individuals, such as variation among patients, may occur.

MiRNA expression profiling and mRNA expression profiling analyses from both GEO and TCGA STAD datasets were carried out. This resulted in 1042 mRNAs and 79 miRNAs differentially expressed. Since miRNAs tend to negatively regulate their target genes, the expression profiles of the miRNA and mRNA should be inversely correlated. Combining an integrated analysis using inverse co-relationship between these differentially expressed miRNAs and mRNAs and three prediction tools (miRanda, PITA and RNAhybrid), 3206 miRNA–mRNA pairs were obtained.

Aberrant expression of miRNA might play significant roles in the pathogenesis and prognosis of GC [26]. As down-regulated miRNAs act as tumor suppressors miRNAs in cancer, attention was made to select down-regulated miRNAs for further studies. For the preliminary screening of miRNAs, three down-regulated miRNAs, miR-30a, miR-26a and miR-204 which were also previously reported to be down-regulated in many different cancers including gastric cancer were selected and subjected to qRT-PCR analysis. MiR-30a had a tumor suppressor role in non-small cell lung cancer [27], colon cancer [28] and breast cancer [29]. MiR-30a is closely associated with relapse-free survival among patients with gastric cancer [30]. MiR-26a was down-regulated and functions as a tumor suppressor in gastric cancer [31], breast cancer [32], nasopharyngeal carcinoma [33] and hepatocellular carcinoma [34]. MiR-204 was found to be deregulated in gastric cancer [35] and its down-regulation causes gastric cancer tumorigenesis by Ras activation [36] which is one of the most well-known oncogenes. Oncogenic *Bcl-2* is activated by the inactivation of miR-204, thus supporting its tumor suppressor role in GC [37]. Also, miR-204 acts as a tumor suppressor by targeting eighteen genes in head and neck squamous cell carcinoma [38]; miR-204 is deregulated and plays a crucial role in endometrial cancer progression by targeting *FOXC1* [19]. Genomic loci encoding miR-204 are frequently lost in various cancers such as ovarian, breast and pediatric renal cancer, and this micro-deletion is directly associated with dysregulation of important oncogenic pathways which play significant roles in tumorigenesis and metastasis [20]. Consistent with previous studies, among the three selected miRNAs, the expression of miR-204 was found to be lowest in AGS cells, so miR-204 was further selected for in vitro studies in AGS cells. In this study, miR-204 was ectopically over-expressed and a cell functional assay was performed which showed that miR-204 suppressed the proliferation of gastric cancer cells.

Dysregulation of mRNA contributes to the tumorigenesis of GC [39]. To profile the target genes when miR-204 was over-expressed, we performed RNA-seq analysis. Five of the common miR-204 targets namely *CD55*, *CKS1B*, *CXCL1*, *GPRC5A* and *TNS4* were selected among the 29 target genes both from RNA-seq and three target prediction tools (miRanda, PITA and RNAhybrid) for experimental validation. Gene ontology analysis revealed that these target genes have roles in different cell functions such as cell proliferation, regulation of cell cycle, cell cycle, signal transduction, etc. The over-expression of these five target genes were also reported in different tumorigenesis events and considered as important target genes. Even though many studies highlighted the interaction of miR-204 and its target genes, the interaction between the selected candidate target genes and miR-204 are still not validated in GC. An extensive literature search revealed that these target genes play potential roles in gastric cancer.

The rationales for selecting these target genes are discussed here. *CD55* is a member of membrane-bound complement-regulatory proteins and could be useful as molecular markers for prognosis and therapy of gastric carcinoma patients [40]. *CD55* overexpression has been found in breast [41] and gall bladder cancer [42]. *CKS1B* is also associated with lymph node metastasis and is associated with poor prognosis in gastric adenocarcinoma [43]. The *CKS1B* gene is located at chromosome 1q21, which is over-represented in hepatocellular [44], and nasopharyngeal carcinoma [45]. *CXCL1* is a chemokine that has roles in development, homeostasis and the immune system [46] and its expression was significantly higher in serum samples of gastric cancer patients in comparison to healthy donors. *CXCL1* up-regulation may contribute to both the development and progression of hepatocellular carcinoma [47]. Global gene expression profiling revealed that *GPRC5A* was significantly elevated in gastric cancer and may act as a potential biomarker [48]. *GPRC5A* is over-expressed in colon cancer [49] and hepatocellular carcinoma [50]. *TNS4* is the member of the tensin family [51] and has oncogenic activity in colon cancer [52], thymoma [53] and lung cancer [54]. There is frequent over-expression of *TNS4* in gastric cancer showing the biologically aggressive behavior with its high expression, so high *TNS4* mRNA expression may be a novel prognostic predictor for gastric cancer patients [55]. The results of expression profiles of these five target genes from our analysis are concordant with the previous studies.

In this study, using the expression profiles of miRNAs and mRNAs with dysregulated expression in gastric cancer compared with those in normal controls and by combining the interactions between the up-regulated mRNAs and down-regulated miRNAs, possible regulatory relationships between miRNAs and mRNAs in gastric cancer were revealed. The results obtained from experiments like cell functional assays and qRT-PCR provide the insight that miR-204 might play a tumor suppressor role in gastric cancer by regulating five of its targets, *CD55*, *CKS1B*, *CXCL1*, *GPRC5A* and *TNS4*. Finally, luciferase reporter assays indicate that miR-204 might target *CKS1B*, *CXCL1* and *GPRC5A* via their 3′UTR. The regulation of these target genes by miR-204 was not reported previously and the results of this study provide the first evidence of regulation of these target genes by miR-204. The results of this study uncover the potential role of miR-204 as a tumor suppressor in gastric cancer and, therefore, acts as a useful potential therapeutic agent in the treatment of gastric cancer.

## 4. Materials and Methods

### 4.1. Datasets Collection

Four microarray expression profile datasets for mRNA, GSE13911 [56], GSE19826 [57], GSE17187 [58], and GSE22377 [59] were obtained from GEO which contained 99 gastric cancer samples and 46 normal samples. Three microarray expression profile datasets for miRNA, GSE33743 [60], GSE30070 [35] and GSE23739 [61] were obtained from GEO which contained 167 gastric cancer samples and 78 normal samples.

The mRNA expression datasets from TCGA STAD which contained 238 gastric cancer samples and 33 normal samples from the Illumina sequencing platform were collected. Similarly, the miRNA expression datasets from TCGA STAD which contained 240 gastric cancer samples and 30 normal samples from the Illumina sequencing platform were also collected.

### 4.2. Expression Profiling Data Analysis

The collected mRNA and miRNA datasets were analyzed using R statistical software package (http://www.R-project.org). Gene Chip Robust Multichip Average (GCRMA) was used for normalization which can adjust the background intensity and normalize the probe intensity of Affymetrix data. A *p*-value <0.05 was considered as significant and log2 fold change value was set as the criteria to screen out differentially expressed mRNA and miRNA. Log2 fold change greater or less than ±1 for mRNA and log2 fold change greater or less than ±0 for miRNA was adjusted as filter to find differentially expressed miRNAs.

### 4.3. Identifying Target Genes by Inverse Correlation and Target Prediction

Pearson’s correlation analysis was applied to the differentially expressed miRNAs and differentially expressed mRNAs. Since miRNAs act as negative regulators, up-regulated miRNAs resulted in down-regulated target mRNAs, and vice versa. Four target prediction tools miRanda [16], PITA [17], RNAhybrid [18], and TargetScan [62] were used to find the potential miRNA and target gene interactions.

### 4.4. Functional Enrichment Analysis

Functional enrichment analysis of miRNA target genes was carried out using DAVID [63] and analyzed in context of gene ontology (GO) and Kyoto Encyclopedia of Genes and Genomes (KEGG) biological pathway using the molecular annotation [64]. The genes were assigned to functional groups based on molecular function, biological processes, and specific pathways.

### 4.5. Cell Line and Culture Condition

Human gastric cancer cell line AGS was purchased from BCRC, Taiwan. The AGS cell line was cultured in ATCC formulated Ham’s F-12K medium supplemented with 10% fetal bovine serum. The culture medium was supplemented with 1% penicillium/streptomycin antibiotic. Cells were maintained at 37 °C in a humified atmosphere in the presence of 5% CO_2_. All medium and related reagents were purchased from Invitrogen.

### 4.6. Total RNA Isolation

The AGS gastric cancer cell lines were grown to about 70% confluency and the growing medium was sucked off and the cells were collected in the Eppendorf tube. Total RNA was isolated using TRIzol kit (Ambion, Foster City, CA, USA). Briefly, 1 mL of TRIzol LS reagent was added and pipetted well to mix to make the homogenate mixture. The tube containing the homogenate was allowed to incubate at room temperature for 10 min. 100 μL of chloroform was added to the tube and shook vigorously for 30 s and kept at room temperature for 10 min and then centrifuged at 12,000× *g* at 4 °C for 15 min. Then 600 μL upper aqueous phase was transferred to the new reaction tube and 500 μL cold isopropanol (−20 °C) was added and kept at room temperature for 10 min and centrifuged at 12,000× *g* at 4 °C for 30 min. The supernatant was removed and 1 mL of 70% ethanol was used for washing. After washing, it was centrifuged at 7500× *g* for 5 min at 4 °C spin and the supernatant was removed. The pellet was air dried and eluted with 20–30 μL of RNase free water and then incubated in a water bath set at 55 °C. The quantity of total RNA was measured by Nanodrop Spectrophotometer (Thermo Fisher Scientific, Waltham, MA, USA) at absorbance 260 nm by calculating A260/A230 and A260/A280 ratios.

### 4.7. Ectopic Over-Expression of miR-204

The miR-204-5p mimic (5′UUCCCUUUGUCAUCCUAUGCCU3′) and scrambled control were purchased and prepared according to manufacturer instructions (Dharmacon, Lafayette, CO, USA). For transfection, 4 × 10^5^ cells per well in a volume of 2 mL were grown in 6 well plates one day before transfection and incubated at 37 °C and 5% CO_2_. 80 nM of miR-204 mimic and scrambled control were introduced into the cell lines by transfection using Turbofect (Dharmacon) Reagent. The cells were harvested after 48 h transfection for mRNA and miRNA analyses.

### 4.8. Reverse Transcription of Total RNA Containing miRNA and mRNA

Both mRNA and miRNA were reverse transcribed to cDNA from total RNA. For reverse transcription of total RNA containing miRNA miScript II RT Kit (Qiagen, Hilden, Germany) was used. Briefly, 5× miScript HiSpec Buffer 4 μL, 10× miScript Nucleics Mix 2 μL, miScript Reverse Transcriptase Mix 2 μL, Template RNA 1 μg and variable amount of RNase-free water to make total reaction volume 20 μL was incubated in PCR tube for 60 min at 37 °C and 5 min at 95 °C to inactivate miScript Reverse Transcriptase Mix.

For reverse transcription of total RNA containing mRNA, GScript First-Strand Synthesis (GeneDirex, New Taipei City, Taiwan) protocol was used. For reverse transcription, 1 μL of oligo, 2 μg of template RNA, 1 μL of 10 mM dNTP mix and variable amount of RNase free water were mixed to make total volume 13 μL. Then the mixture was incubated at 65 °C for 5 min and then placed on ice for 1 min. To the same Eppendorf tube, 4 μL of 5× First Strand Buffer, 1 μL of 0.1 M DTT, 1 μL of DEPC water, 1 μL of GScript RTase were added and pipetted gently up and down and incubated at room temperature for 5 min. The mixture was then incubated at 50 °C for 60 min and inactivated by heating at 70 °C for 15 min. The cDNA obtained was stored at −20 °C.

### 4.9. Quantitative Real-Time PCR for miRNA

Quantitative real-time quantitative PCR (qRT-PCR) was performed using standard protocols on Lightcycler 480 real-time PCR system (Roche, Basel, Switzerland). The expression of mature miRNA was measured by qRT-PCR with miScript SYBR Green PCR Kit (Qiagen). The miRNA specific primer for miR-204 was 5′UUCCCUUUGUCAUCCUAUGCCU3′; miR-26a 5′UUCAAGUAAUCCAGGAUAGGCU3′, and miR-30a 5′UGUAAACAUCCUCGACUGGAAG3′. For qRT-PCR of miRNA, 2× QuantiTect SYBR Green PCR Master Mix 10 μL, 10× miScript Universal Primer 2 μL, 10× miScript Primer Assay 2 μL, 200 ng template cDNA and variable amount of RNA free water were mixed to make total reaction volume 20 μL. The reaction mix was incubated for 15 s at 95 °C for PCR initial activation step, denaturation at 94 °C for 15 s, annealing at 55 °C for 30 s and extension at 70 °C for 30 s with 45 cycles. miRNA expression level was normalized to U6 miRNA and calculated from the triplicates of *C*_t_ values using the ΔΔ*C*t method.

### 4.10. Quantitative Real-Time PCR for mRNA

The expression of potential target genes of miRNA was measured by qRT-PCR with SYBR Green PCR Kit (Roche). For qRT-PCR of mRNA, SYBR Master mix 5 μL, Forward primer 1 μL, Reverse primer 1 μL, 50 ng cDNA and variable amount of RNA free water were mixed to make total reaction volume 10 μL. The reaction mix was incubated at 95 °C for PCR initial activation step, denaturation at 95 °C for 15 s, annealing at 60 °C for 30 s and extension at 72 °C for 30 s. The expression level of mRNA was calculated by threshold cycle *C*t and the relative expression level was calculated using ΔΔCt method. The relative expression levels of mRNA were normalized to GAPDH. The primers sequences for *CD55* forward 5′-AGGCATTTTCATCTTTCCTTCGGG-3′ and reverse 5′-CCTTATCACCATCAACACCCCTGG-3′ [65]; *CKS1B* forward 5′-GGACAAATACGACGACGAGGA-3′ and reverse 5′-CTGACTCTGCTGAACGCCAAG-3′ [44]; *CXCL1* forward 5′-ATGGCCCGCGCTGCTCTCTCC-3′ and reverse 5′-GTTGGATTTGTCACTGTTCAG-3′ [66]; *GPRC5A* forward 5′-GCTGCTCACAAAGCAACGAA-3′ and reverse 5′-ATAGAGCGTGTCCCCTGTCT-3′ [67]; *TNS4* forward 5′-AGAGAACTGGGAGGTGCAGA-3′ and reverse 5′-AGTCAGAGTGATGCCCTGCT-3′ [68].

### 4.11. RNA Sequencing

Total RNA was isolated from the AGS cells transfected with the miR-204 mimic and scrambled control for library preparation which involved a series of steps to convert the input RNA into small fragments of DNA that could be sequenced by the Illumina machine (Next-seq 500 sequencing system, Illumina, San Diego, CA, USA). All the protocols from Illumina were followed for RNA sequencing. In brief, mRNA-seq library preparation protocol included poly-A RNA isolation, RNA fragmentation, reverse transcription to cDNA using random primers, 3′ends adenylation, adapter ligation and PCR enrichment. The resulting cDNA library was measured quantitatively by qRT-PCR and Qubit Fluorometry. Then it was placed in one of the eight lanes of a flow-cell. Individual cDNA fragments attach to the surface of the lane and subsequently undergo an amplification step, whereby they were converted into clusters of double-stranded DNA. The flow cell was then placed in the Illumina NextSeq 500, where each cluster was sequenced in parallel. Specifically, at each cycle, the four fluorescently labeled nucleotides were added and the signals emitted at each cluster were recorded. For each flow-cell, this process was repeated for a given number of cycles (single end, 75 base-pairs). The analysis flow for RNA-seq data has four steps; quality control, aligning to reference genome, discovering splice junction and transcript assembly, estimates their abundance and normalization.

### 4.12. Real-Time Cell Proliferation Assay

The cell proliferation assay was performed using the xCELLigence RTCA DP (Real-time cell growth analysis dual plate) instrument (ACEA Biosciences, San Diego, CA, USA). The RTCA is an impedance-based technology that allows for label-free and real-time monitoring of cellular phenotypic changes like cell adherence, proliferation and migration in real time. The background was measured in wells containing 100 μL of cell culture medium without cells for 30 s. The AGS cells from each well were detached from the flasks by a brief treatment with trypsin/EDTA, re-suspended in cell culture medium. The day before transfection, 5000 cells per well were seeded to 16 well E-plates, each well containing 100 μL of culture medium. Transfection was carried out using 80 nM miR-204 mimic or 80 nM scrambled control using Turbofect transfection reagent. The E-plates were incubated at room temperature for 30 min and placed on the plate reader in the incubator for continuous impedance recording. All procedures were performed in the laminar flow cabinet. The cells were real-time monitored at 37 °C in humidified 5% CO_2_ atmosphere. The cell proliferation data were recorded every 15 min for 96 h. Wells containing DMEM were used as negative control for impedance baseline measurement. In all the experiments, the final volume in the micro-wells was 200 μL. The maximum cell index (CI max) was calculated as the highest CI observed during the study.

### 4.13. Selection of Potential Target Genes of miRNA for Experimental Validation

For selection of candidate target genes of miR-204, all the predicted target genes of miR-204 predicted by three prediction tools; miRanda, PITA and RNAhybrid and all the down-regulated target genes from RNA-seq analysis were intersected. Next, screening of the common target genes was done with annotated biological functions that could be related to regulation of cell cycle, regulation of cell proliferation, cell death, cell cycle, programmed cell death, regulation of apoptosis, anti-apoptosis, signal transduction and immune response, etc. Using above approach, five candidates, *CD55*, *CKS1B*, *CXCL1*, *GPRC5A* and *TNS4* were selected for further analysis.

### 4.14. Plasmid Construction and Dual Luciferase Reporter Assay

The 3′UTR of five target genes *CD55*, *CKS1B*, *CXCL1*, *GPRC5A* and *TNS4* were amplified using genomic DNA and cloned downstream of the Renilla luciferase open reading frame in the pmiRGLO vector (Promega, Madison, WI, USA) using PmeI and XbaI restriction sites. The primers for 3′UTR amplification are shown in Table 5.

Day before performing the luciferase reporter assay, 12 well plate with 1 × 10^4^ AGS cells were co-transfected with either empty luciferase vector or the construct containing 3′UTR of five target genes, *CD55*, *CKS1B*, *CXCL1*, *GPRC5A* and *TNS4* and miR-204 mimic or scrambled control. pmiRGLO vector with no insert was used as control. The plates were removed from the incubator next day and 200 μL of Dual-Glo reagent was added to each well and mixed and allowed to wait for 10 min for cell lysis to occur, then firefly luminescence was measured using Lumat 9507 LB (Berthold Technologies, Oak Ridge, TN, USA). 100 μL of Dual-Glo Stop and Glo was added to the cell lysate and mixed well and then renilla luminescence was measured. Normalized luciferase activity (firefly luciferase activity/renilla luciferase activity) for each construct was calculated.

### 4.15. Statistical Analysis

All the expression profiling data analysis was done using R Statistical Software Package. *p*-value less than 0.05 was considered statistically significant. All the experiments were performed in at least three times and the values represent the mean of triplicate samples and standard errors.

## Figures and Tables

**Table 1:** Differentially expressed miRNAs from GEO and TCGA STAD datasets.

Datasets	Up-Regulated miRNAs	Down-Regulated miRNAs
GSE33743	173	56
GSE30700	94	72
GSE23739	333	173
TCGA STAD	250	108

**Table 2:** Down-regulated miRNAs in at least three datasets.

Datasets	miRNA	Log_2_FC	Datasets	miRNA	Log_2_FC	Datasets	miRNA	Log_2_FC
TCGA STAD	miR-204-5p	−1.82	GSE23739	miR-204-5p	−1.69	GSE30700	miR-375	−1.74
	miR-145-5p	−1.24		miR-375	−1.35		miR-204-5p	−1.35
	miR-375	−0.86		miR-145-5p	−1.35		miR-642a-5p	−0.75
	miR-642a-5p	−0.79		miR-642a-5p	−1.31		miR-26a-5p	−0.47
	miR-26a-5p	−0.68		miR-26a-5p	−0.88		miR-145-5p	−0.46
	miR-30a-5p	−0.42		miR-342-3p	−0.71		miR-342-3p	−0.14
	miR-342-3p	−0.04		miR-30a-5p	−0.53		miR-30a-5p	−0.09
TCGA STAD	miR-1-3p	−1.86	GSE33743	miR-1-3p	−2.49	GSE30700	miR-1-3p	−1.36
	miR-203a-3p	−1.03		miR-203a-3p	−1.14		miR-203a-3p	−0.96

**Table 3:** Important gene ontology terms for miR-204 down-regulated targets.

Term	Count	*p*-Value
Cell cycle	88	1.97 × 10^−4^
Cell cycle process	66	6.38 × 10^−4^
Cell cycle phase	51	9.06 × 10^−4^
Cell division	38	0.002
Cell proliferation	50	0.004
Programmed cell death	61	0.028
Apoptosis	60	0.030

**Table 4:** Gene ontology of selected target genes of miR-204.

Target Genes	Gene Ontology
*CD55*	Regulation of complement activation, innate immune response
*CKS1B*	Cell cycle, cell proliferation, cell division
*CXCL1*	Negative regulation of cell proliferation, cell proliferation, inflammatory response, signal transduction, immune response
*GPRC5A*	Signal transduction
*TNS4*	Cell death, programmed cell death, apoptosis

**Table 5:** Primers for 3′UTR cloning.

Target Gene	Sequence(5′-3′)
*CD55-UCSC F*	GTTTAAACCCAAAGAAGAGTTAAGAAGAAAATACACACAAGTAT
*CD55-UCSC R*	TGCTCTAGATTAAGGAGGAAAAAAAGTTTTATTTTAAGAAATACACATTAAA
*CKS1B-UCSC F*	GTTTAAACAGCTGGCAAGCTACTTTTCAGC
*CKS1B-UCSC R*	TGCTCTAGATAGATTATAAAAACTTCCTCTTTAATCAAGGCTTTTAACATG
*CXCL1-UCSC F*	GTTTAAACCCAGAAGGGAGGAGGAAGCTC
*CXCL1-UCSC R*	TGCTCTAGATATAAATCACCAGATTTTCCAGTAAAGGTAGCC
*GPRC5A-UCSC F*	GTTTAAACCTCTGTCCTGAAGAGTGGGACA
*GPRC5A-UCSC R*	TGCTCTAGATTACTGGTAACTGCTGCCACC
*TNS4-UCSC F*	GTTTAAACGGGAGAGACTGCCTGTGC
*TNS4-UCSC R*	TGCTCTAGATTACTGTTTTGCAAAGACAAACATTTTATTTTTCATGA

## Supplementary Materials

The following are available online at www.mdpi.com/1422-0067/19/1/87/s1.
