# Supplementary material for: Integrated MicroRNA–mRNA Analysis Reveals miR-204 Inhibits Cell Proliferation in Gastric Cancer by Targeting CKS1B, CXCL1 and GPRC5A
Source: Int J Mol Sci. 2017 Dec 28;19(1):87. doi: 10.3390/ijms19010087 (PMC5796037; doi:10.3390/ijms19010087)
Supplement: Supplementary file 1 [file ijms-19-00087-s001.zip › ijms-248236-supplementary/supplementary materials.pdf]

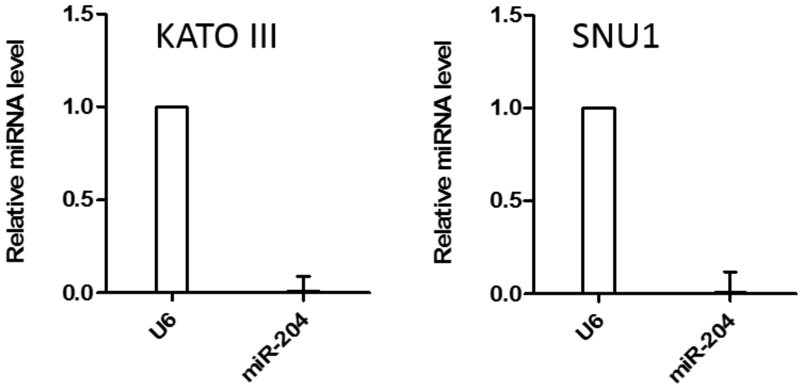

Supplementary Figure S1. Relative miR-204 expression in KATO III and SNU1 cell lines. miRNA expression level was normalized to U6 miRNA and calculated from the triplicates data.

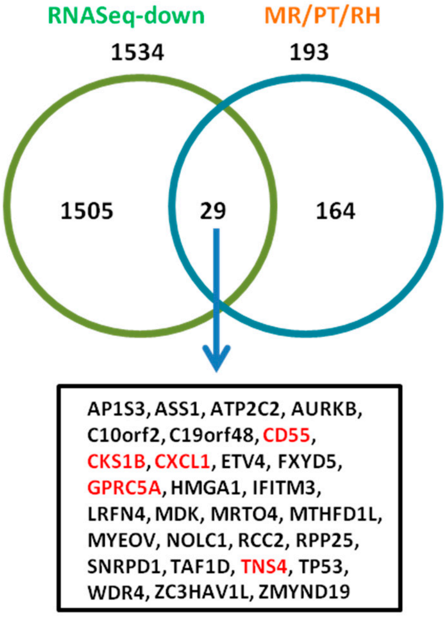

Supplementary Figure S2. Venn diagram showing the intersection of genes obtained from RNA-seq analysis that were down-regulated when miR-204 was over-expressed in AGS cells and the target of miR-204 predicted by miRanda, PITA and RNAhybrid.

Additional file 1: **Table S1.** Up-regulated genes in gastric cancer in GPL570. **Table S2.** Down-regulated genes in gastric cancer in GPL570. **Table S3.** Up-regulated genes in gastric cancer in TCGA STAD datasets. **Table S4.** Down-regulated genes in gastric cancer in TCGA STAD datasets. **Table S5**. Differentially expressed genes in gastric cancer that were common in GPL570 and TCGA STAD datasets.

Additional file 2: **Table S1.** Up-regulated miRNAs in gastric cancer in GSE33743. **Table S2.** Down-regulated miRNAs in gastric cancer in GSE33743. **Table S3.** Up-regulated miRNAs in gastric cancer in GSE30070. **Table S4.** Down-regulated miRNAs in gastric cancer in GSE30070. **Table S5**. Up-regulated miRNAs in gastric cancer in GSE23739. **Table S6**. Down-regulated miRNAs in gastric

cancer in GSE23739. **Table S7**. Up-regulated miRNAs in gastric cancer in TCGA STAD datasets. **Table S8**. Down-regulated miRNAs in gastric cancer in TCGA STAD datasets. **Table S9**. Differentially expressed miRNAs in gastric cancer in at least three datasets.

Additional file 3: Table S1. Total miRNA target genes interactions from correlation analysis and four target prediction tools. **Table S2**. Total miRNA target genes interactions predicted by miRanda, PITA and RNAhybrid.

Additional file 4: Table S1. Gene ontology analysis of miRNA target genes predicted by three prediction tools; miRanda, PITA and RNAhybrid.

Additional file 5: Table S1. Up-regulated target genes from RNA-seq when miR-204 was over-expressed. **Table S2**. Down-regulated target genes from RNA-seq when miR-204 was over-expressed.

Additional file 6: **Table S1**. miR-204-5p targets in miRTarBase.
